# Feasibility and Safety of Intracardiac Echocardiography Use in Transcatheter Left Atrial Appendage Closure Procedures

**DOI:** 10.1016/j.jscai.2022.100510

**Published:** 2022-11-03

**Authors:** Salman Zahid, Smitha Gowda, Anas Hashem, Muhammad Zia Khan, Waqas Ullah, Gurleen Kaur, Usama Nasir, Devesh Rai, Nadeen N. Faza, Stephen H. Little, Miguel Valderrabano, Sachin S. Goel

**Affiliations:** a Department of Medicine, Rochester General Hospital, Rochester, New York; b Department of Cardiology, Houston Methodist DeBakey Heart and Vascular Center, Houston, Texas; c Division of Cardiovascular Medicine, West Virginia University Heart and Vascular Institute, Morgantown, West Virginia; d Department of Cardiovascular Medicine, Jefferson University Hospitals, Philadelphia, Pennsylvania; e Department of Medicine, Brigham and Women's Hospital, Boston, Massachusetts; f Department of Medicine, Reading Hospital and Medical Center, Reading, Pennsylvania

**Keywords:** echocardiography, intracardiac echocardiography, left atrial appendage closure

## Abstract

**Background:**

Left atrial appendage closure (LAAC) is usually performed under the guidance of transesophageal echocardiography (TEE). Data on the safety of intracardiac echocardiogram (ICE)–guided LAAC from a real-world population in the United States remain limited. In this study, the aim was to evaluate the trends and outcomes of ICE-guided LAAC procedures using the US National Inpatient Sample.

**Methods:**

This study used the National Inpatient Sample database from quarter 4 of 2015 to 2019. We used a propensity-matched analysis and adjusted odds ratios for in-hospital outcomes/complications. A *P* value of <.05 was considered significant.

**Results:**

We identified 61,995 weighted LAAC cases. Of these, 1410 patients had ICE-guided LAAC with a lower median age than the patients who had TEE-guided LAAC (75 vs 77 years; *P* ≤ .01). The use of ICE-guided LAAC increased from 1.7% in 2015 to 2.2% in 2019 (*P*_trend_ = .75). Major, cardiovascular, neurologic, and pulmonary complications were similar for ICE-guided and TEE-guided LAAC on adjusted analysis. On propensity-matched analysis, the overall vascular complication rates were similar. However, retroperitoneal bleeding remained significantly higher (0.7% vs 0%) with ICE. Gastrointestinal bleeding complications were more frequent in TEE-guided LAAC (3.5% vs 2.1%). The length of stay was similar for both groups (median = 1 day; *P* = .23); however, ICE was associated with $1769 excess cost of hospitalization ($25,112 vs $23,343; *P* = .04).

**Conclusions:**

ICE–guided LAAC is safer than TEE-guided LAAC, with similar rates of major complications. However, ICE use was associated with lower rates of gastrointestinal bleeding and higher rates of retroperitoneal bleeding. In addition, ICE-guided LAAC is associated with a similar length of stay but higher costs of hospitalization.

## Highlights

• Intracardiac echocardiographyis safe with similar rates of major complications
• Rates of vascular complications are higher with Intracardiac echocardiography
• Intracardiac echocardiography is associated with higher hospitalization cost

## Introduction

Left atrial appendage closure (LAAC) has emerged as an alternative to long-term anticoagulation for stroke prevention in patients with nonvalvular atrial fibrillation.^1^, ^2^, ^3^ In the United States, most centers perform transesophageal echocardiography (TEE)–guided and fluoroscopy-guided LAAC procedures. TEE has remained the gold standard; however, intracardiac echocardiography (ICE) has emerged as an alternative imaging modality in some centers. Currently, in the United States, ICE guidance is used routinely for catheter-based ablation for arrhythmias and transcatheter closure of patent foramen ovale and atrial and ventricular septal defects. However, data on the safety and feasibility of ICE in LAAC remain limited to small observational studies, the majority of which were performed outside the United States.^4^, ^5^, ^6^, ^7^, ^8^ In this study, we aimed to evaluate trends and outcomes of ICE use in LAAC procedures from a real-world population in the US National Inpatient Sample (NIS).

## Methods

This study used data from the NIS database from the quarter 4 of 2015 to 2019. The NIS is one of the several databases managed by the Agency for Healthcare Research and Quality through a Federal-State-Industry partnership called the Healthcare Cost and Utilization Project (HCUP).^9^ The NIS contains administrative claims data from more than 7 million inpatient hospitalizations annually in 47 participating states plus the District of Columbia, representing >97% of the US population. Because NIS data are compiled annually, the data can be used for the analysis of procedure trends over time using trend weights compiled by the HCUP. For the cost of care, charge to cost ratio supplied by HCUP derived from the Centers for Medicare and Medicaid Services was applied to the total hospital charges. Institutional review board approval and informed consent were not required for this study because NIS data are deidentified and publicly available. As per the HCUP guidelines, observations with cell counts of <11 are reported as “<11.”

**Figure 1:**
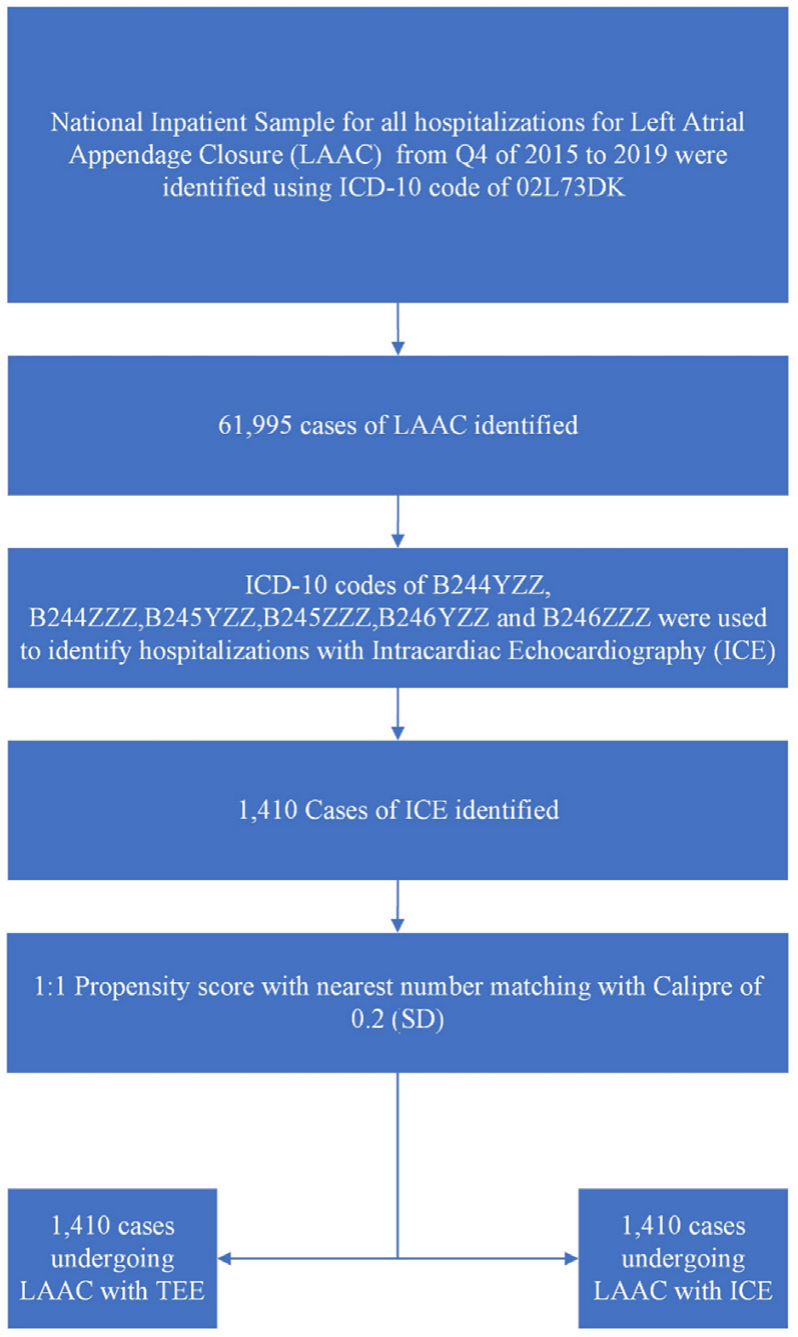
**Study flow chart.** ICD-10, *International Classification of Diseases*, *Tenth Revision*; ICE, intracardiac echocardiography; LAAC, left atrial appendage closure; Q4, quarter 4; TEE, transesophageal echocardiography.

We analyzed NIS data using the *International Classification of Diseases*, *Tenth Revision*, *Clinical Modification* (ICD-10-CM) claim codes. ICD-10-CM code 02L73Dk was used to identify patients undergoing LAAC performed under the guidance of TEE. To identify ICE use, we used the *International Classification of Diseases*, *Tenth Revision*, codes of B244ZZZ, B245YZZ, B245ZZZ, B246YZZ, and B246ZZZ. All diagnosis and procedure fields were queried to select and categorize the study population. Individuals aged <18 years were excluded from the study. A detailed methods flow sheet and study summary is presented in Figure 1 and the Central Illustration.

**Central Illustration:**
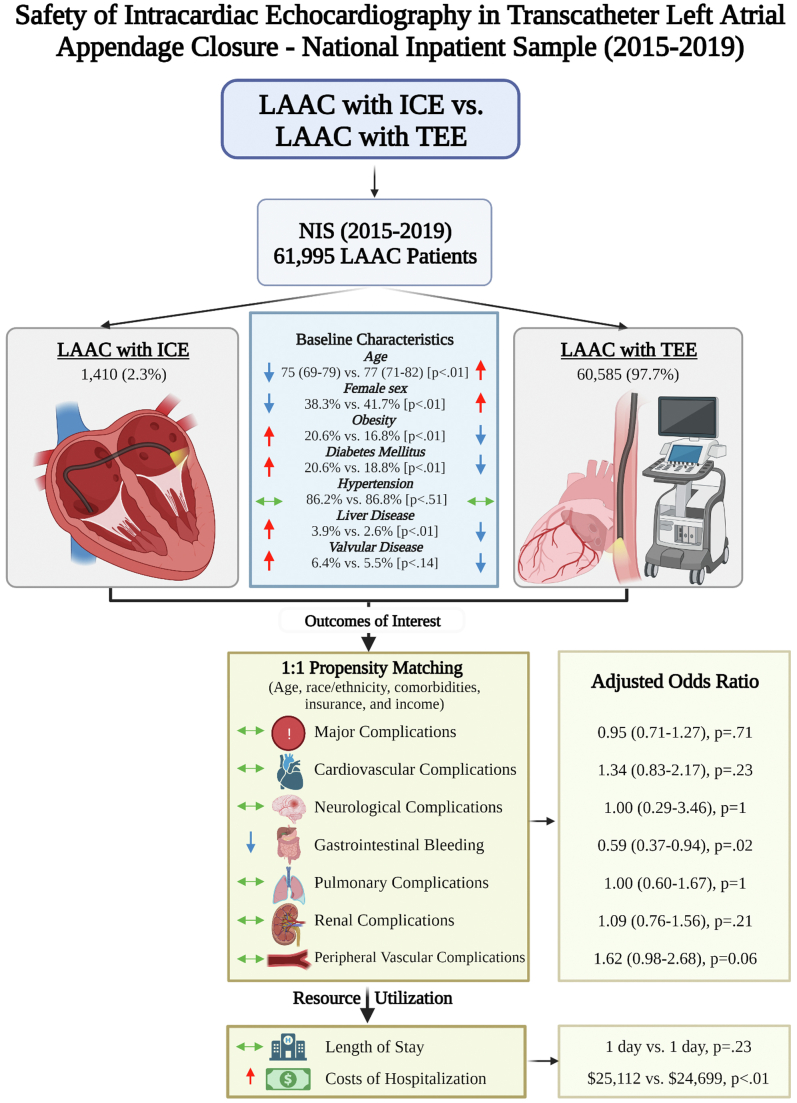
Safety of ICE in transcatheter LAAC: NIS (2015-2019). ICE, intracardiac echocardiography; LAAC, left atrial appendage closure; NIS, National Inpatient Sample; TEE, transesophageal echocardiography.

The primary study end point was major complications. Major complications are defined as the composite of pericardial effusion requiring intervention, cardiac arrest, ischemic stroke/transient ischemic attack, hemorrhagic stroke, any bleeding, myocardial infarction, and peripheral vascular complications, which included arteriovenous fistula, pseudoaneurysm, access site hematoma, retroperitoneal bleeding, and venous thromboembolism. The secondary end points included the following: (1) any cardiovascular complications, (2) peripheral vascular complications, (3) neurologic complications, (4) gastrointestinal (GI) or hematologic complications, (5) pulmonary complications, (6) renal complications, and (7) resource utilization (length of stay [LOS] and cost of hospitalization). Associated procedures and complications were identified using the ICD-10-CM codes (Supplemental Table S1). The definitions of primary and secondary outcomes are shown in Supplemental Table S2.

**Figure 2:**
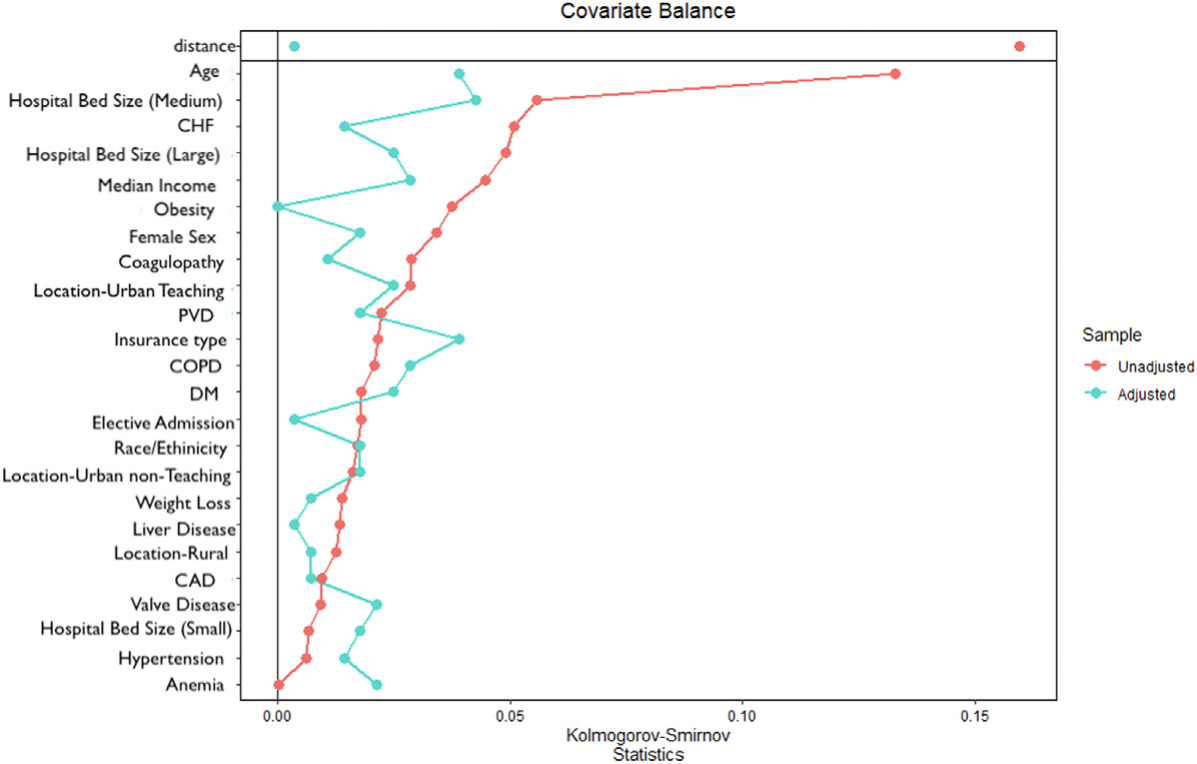
**Balance of covariates before and after propensity matching.** CAD, coronary artery disease; CHF, congestive heart failure; COPD, chronic obstructive pulmonary disease; DM, diabetes mellitus; PVD, peripheral vascular disease.

To account for potential confounding factors and selection bias, a propensity score–matching model was developed using logistic regression to derive 2 matched groups for comparative outcomes analysis. A nearest-neighbor 1:1 variable ratio, parallel, balanced propensity-matching model was made using a caliper width of 0.2 SD (Supplementary Figures 1-4). Baseline characteristics and outcomes before and after propensity match are shown in Figure 2, Tables 1 and 2. Descriptive statistics were presented as frequencies with percentages for categorical variables and as medians with interquartile ranges for continuous variables. Baseline characteristics were compared using the Pearson χ^2^ and Fisher exact tests for categorical variables and Mann-Whitney *U* test for continuous variables. A *P* value of slope was used to assess temporal trends. The baseline characteristics in the propensity-matched samples were compared using standardized mean difference. The in-hospital outcomes for dichotomous variables were compared using odds ratios (ORs) on Cochran–Mantel–Haenszel test. Data were complete for all variables, except for race (n = 1890, 3%), primary payer (n = 70, 0.1%), and median household income (n = 865, n = 1.4%). Given that the overall missing values were low (<3%), they were assumed to be missing at random. The missing values were recoded as “others” before propensity matching. For all analyses, a 2-tailed *P* value of <.05 was considered statistically significant. All statistical analyses were performed using the SPSS version 27 (IBM Corporation) and R version 4.5 (R Foundation for Statistical Computing), for propensity-matched analyses.^10^ Because of the complex survey design of the NIS, sample weights, strata, and clusters were applied to the raw data to generate national estimates for all analyses.

**Table 1:** Baseline characteristics of the study population.

Variable	All patients undergoing LAAC			Propensity-matched sample 1:1		
	TEE-guided (n = 60,585)	ICE-guided (n = 1410)	*P* value	TEE-guided (n = 1410)	ICE-guided (n = 1410)	Standardized mean difference
Age, y	77 (71-82)	75 (69-79)	<.01	75 (70-80)	75 (69-79)	0.018
Women	25,270 (41.7)	540 (38.3)	.01	515 (36.5)	540 (38.3)	0.037
Race	–	–	.05	–	–	0.091
White	51,305 (87.3)	1170 (85.4)	–	1165 (82.6)	1170 (83.0)	–
Black	2470 (4.2)	60 (4.4)	–	50 (3.5)	60 (4.3)	–
Hispanic	2920 (5.0)	90 (6.6)	–	100 (7.1)	90 (6.4)	–
Asian or Pacific Islander	860 (1.5)	15 (1.1)	–	10 (0.7)	15 (1.1)	–
Native American	195 (0.3)	<11 (<0.8)^a^	–	0 (0.0)	<11 (<0.8)^a^	–
Other	985 (1.7)	30 (2.2)	–	20 (1.4)	30 (2.1)	–
Elective admission	55,505 (91.8)	1265 (90.0)	.02	1275 (90.4)	1265 (89.7)	0.083
Comorbidities	–	–	–	–	–	–
Deficiency anemia	1950 (3.2)	45 (3.2)	.95	15 (1.1)	45 (3.2)	0.148
Congestive heart failure	20,910 (34.5)	415 (29.4)	<.01	395 (28.0)	415 (29.4)	0.031
Chronic pulmonary disease	13,285 (21.9)	280 (19.9)	.06	240 (17.0)	280 (19.9)	0.073
Coagulopathy	2345 (3.9)	95 (6.7)	<.01	110 (7.8)	95 (6.7)	0.041
Coronary artery disease	29,150 (48.1)	665 (47.2)	.48	655 (46.5)	665 (47.2)	0.014
Diabetes	11,370 (18.8)	290 (20.6)	.09	255 (18.1)	290 (20.6)	0.063
Hypertension	52,575 (86.8)	1215 (86.2)	.51	1195 (84.8)	1215 (86.2)	0.040
Liver disease	1555 (2.6)	55 (3.9)	<.01	60 (4.3)	55 (3.9)	0.018
Obesity	10,815 (16.8)	290 (20.6)	<.01	290 (20.6)	290 (20.6)	<0.001
Peripheral vascular disorders	5525 (9.1)	160 (11.3)	<.01	185 (13.1)	160 (11.3)	0.054
Valvular disease	3315 (5.5)	90 (6.4)	.14	60 (4.3)	90 (6.4)	0.095
Weight loss	235 (0.4)	25 (1.8)	<.01	15 (1.1)	25 (1.8)	0.060
Hospital region	–	–	<.01	–	–	0.112
Rural	1180 (1.9)	<11 (<0.8)^a^	–	0 (0.0)	<11 (<0.8)^a^	–
Urban nonteaching	5915 (9.8)	115 (8.2)	–	90 (6.4)	115 (8.2)	–
Urban teaching	53,490 (88.3)	1285 (91.1)	–	1320 (93.6)	1285 (91.1)	–
Hospital bed size	–	–	<.01	–	–	0.010
Small	6420 (10.6)	140 (9.9)	–	165 (11.7)	140 (9.9)	–
Medium	13,805 (22.8)	400 (28.4)	–	340 (24.1)	400 (28.4)	–
Large	40,360 (66.6)	870 (61.7)	–	905 (64.2)	870 (61.7)	–
Primary insurance	–	–	<.01	–	–	0.122
Medicare	53,770 (88.8)	1230 (87.5)	–	1285 (91.1)	1230 (87.2)	–
Medicaid	710 (1.2)	20 (1.4)	–	<11 (<0.8)^a^	20 (1.4)	–
Private insurance	4830 (8.0)	100 (7.1)	–	110 (7.8)	100 (7.1)	–
Self-pay	265 (0.4)	<11 (<0.8)^a^	–	<11 (<0.8)^a^	<11 (<0.8)	–
No charge	30 (0.0)	0 (0.0)	–	0 (0.0)	45 (3.2)	–
Other	915 (1.5)	45 (3.2)	–	1285 (91.1)	1230 (87.2)	–
Median household income (in percentile)	–	–	<.01	–	–	0.086
0-25	12,895 (21.6)	250 (17.9)	–	290 (20.6)	250 (17.7)	–
26-50	15,415 (25.8)	395 (28.3)	–	335 (23.8)	395 (28.0)	–
51-75	16,715 (28.0)	340 (24.4)	–	385 (27.3)	340 (24.1)	–
76-100	14,710 (24.6)	410 (29.4)	–	395 (28.0)	410 (29.1)	–

Values are median (IQR) or n (%). ICE, intracardiac echocardiography; LAAC, left atrial appendage closure; TEE, transesophageal echocardiography.

a As per the Healthcare Cost and Utilization Project regulations, observations with cell counts of <11 are reported as “<11.”

**Table 2:** Hospital encounter outcomes and resource utilization of the study population with intracardiac echocardiography versus transesophageal echocardiography.

Variable	All patients undergoing LAAC			1:1 propensity matching		*P* value
	TEE-guided (n = 60,585)	ICE-guided (n = 1410)	*P* value	TEE-guided (n = 1410)	ICE-guided (n = 1410)	
Died during hospitalization	95 (0.2)	0 (0)	.14	0 (0)	0 (0)	–
Major complications^b^	3375 (5.6)	95 (6.7)	.06	100 (7.1)	95 (6.7)	.71
Any cardiovascular complication	1665 (2.7)	40 (2.8)	.84	30 (2.1)	40 (2.8)	.23
Percutaneous coronary intervention	80 (0.1)	<11 (<0.8)^a^	.03	0 (0.0)	<11 (<0.8)^a^	.03
Cardiac arrest	90 (0.1)	<11 (<0.8)^a^	.05	0 (0.0)	<11 (<0.8)^a^	.03
Heart block	675 (1.1)	<11 (<0.8)^a^	.15	<11 (<0.8)	<11 (<0.8)^a^	.21
ST-elevation myocardial infarction	25 (0.0)	0 (0.0)	.45	–	–	–
Non–ST-elevation myocardial infarction	115 (0.2)	0 (0.0)	.10	<11 (<0.8)^a^	<11 (<0.8)^a^	–
Pericardial effusion requiring intervention	665 (1.1)	25 (1.8)	.02	20 (1.4)	25 (1.8)	.45
Pericarditis	130 (0.2)	<11 (<0.8)^a^	.27	0 (0)	<11 (<0.8)	–
Cardiogenic shock	140 (0.2)	<11 (<0.8)^a^	.07	–	–	–
Any systemic complication	95 (0.2)	<11 (<0.8)^a^	.14	<11 (<0.8)^a^	<11 (<0.8)^a^	.03
Anaphylaxis	25 (0.0)	<11 (<0.8)^a^	.45	–	–	–
Arterial thrombosis	45 (0.1)	<11 (<0.8)^a^	.31	–	–	–
Septic shock	30 (0.0)	<11 (<0.8)^a^	.40	<11 (<0.8)^a^	<11 (<0.8)^a^	.03
Any peripheral vascular complication	770 (1.3)	40 (2.8)	<.01	25 (1.8)	40 (2.8)	.06
AV fistula	130 (0.2)	<11 (<0.8)^a^	.27	0 (0.0)	<11 (<0.8)^a^	.03
Pseudoaneurysm	190 (0.3)	<11 (<0.8)^a^	.01	15 (1.1)	<11 (<0.8)^a^	.32
Hematoma	315 (0.5)	<11 (<0.8)^a^	.41	<11 (<0.8)^a^	<11 (<0.8)^a^	.21
Retroperitoneal bleeding	40 (0.1)	<11 (<0.8)^a^	<.01	0 (0.0)	<11 (<0.8)^a^	<.01
Venous thromboembolism	160 (0.3)	<11 (<0.8)^a^	<.01	<11 (<0.8)^a^	<11 (<0.8)^a^	1
Any neurologic complication	435 (0.7)	<11 (<0.8)^a^	.12	<11 (<0.8)^a^	<11 (<0.8)^a^	1
Hemorrhagic stroke	155 (0.3)	0 (0.0)	.06	–	–	–
Ischemic stroke	130 (0.2)	0 (0.0)	.08	<11 (<0.8)^a^	<11 (<0.8)^a^	.03
Transient ischemic attack	150 (0.2)	<11 (<0.8)^a^	.43	<11 (<0.8)^a^	<11 (<0.8)^a^	.03
Any gastrointestinal or hematologic complication	1500 (2.5)	30 (2.1)	.41	50 (3.5)	30 (2.1)	.02
Gastrointestinal bleeding	1435 (2.4)	30 (2.1)	.56	50 (3.5)	30 (2.1)	.02
Bleeding during the procedure	65 (0.1)	0 (0.0)	.22	–	–	–
Need for blood transfusion	<11 (<0.8)^a^	<11 (<0.8)^a^	–	–	–	–
Any pulmonary complications	1445 (2.4)	30 (2.1)	.53	30 (2.1)	30 (2.1)	1
Respiratory failure	750 (1.2)	<11 (<0.8)	<.01	<11 (<0.8)^a^	<11 (<0.8)^a^	.21
Pneumothorax	<11 (<0.8)^a^	<11 (<0.8)^a^	–	–	–	–
Pleural effusion	235 (0.4)	<11 (<0.8)^a^	.84	0 (0.0)	<11 (<0.8)^a^	.03
Pneumonia bacterial	195 (0.3)	<11 (<0.8)^a^	.83	15 (1.1)	<11 (<0.8)^a^	.03
Need for a ventilator	805 (1.3)	20 (1.4)	.77	<11 (<0.8)^a^	<11 (<0.8)^a^	.07
Renal complications	1475 (2.4)	65 (4.6)	<.01	60 (4.3)	65 (4.6)	.21
Acute kidney injury	1475 (2.4)	65 (4.6)	<.01	60 (4.3)	65 (4.6)	.65
New hemodialysis	55 (0.1)	0 (0.0)	.26	–	–	–
Resource utilization	–	–	–	–	–	–
Length of hospital stay, d,	1 (1-1)	1 (1-1)	<.01	1 (1-1)	1 (1-1)	.23
Median cost of hospitalization, $	24,699 (18,994-31,063)	25,112 (18,487-32,550)	.31	23,343 (18,156-31,235)	25,112 (18,487-32,550)	.04

Values are n (%) or median (IQR). AV, arteriovenous; ICE, intracardiac echocardiography; LAAC, left atrial appendage closure; TEE, transesophageal echocardiography.

a As per the Healthcare Cost and Utilization Project regulations, observations with cell counts of <11 are reported as “<11.”

b Composite of pericardial effusion requiring intervention, cardiac arrest, ischemic stroke/transient ischemic attack, hemorrhagic stroke, any bleeding, myocardial infarction, and peripheral vascular complications, which included AV fistula, pseudoaneurysm, access site hematoma, retroperitoneal bleeding, and venous thromboembolism.

## Results

A total of 61,995 weighted cases of LAAC were identified. Of the included patients, 1410 patients underwent ICE-guided LAAC procedure. Patients in the ICE-guided LAAC group were younger than those in the TEE-guided LAAC group (median age, 75 vs 77 years; *P* = <.01) and had fewer proportion of women (38.3% vs 41.7%; *P* = <.01). With regard to comorbidities, obesity (20.6% vs 16.8%), liver disease (3.9% vs 2.6%), peripheral vascular disease (11.3% vs 9.1%), and coagulopathies (6.7% vs 3.9%) were more frequent in the ICE-guided LAAC group (*P* < .01 for all). The detailed baseline characteristics before and after propensity matching are shown in Table 1.

**Figure 3:**
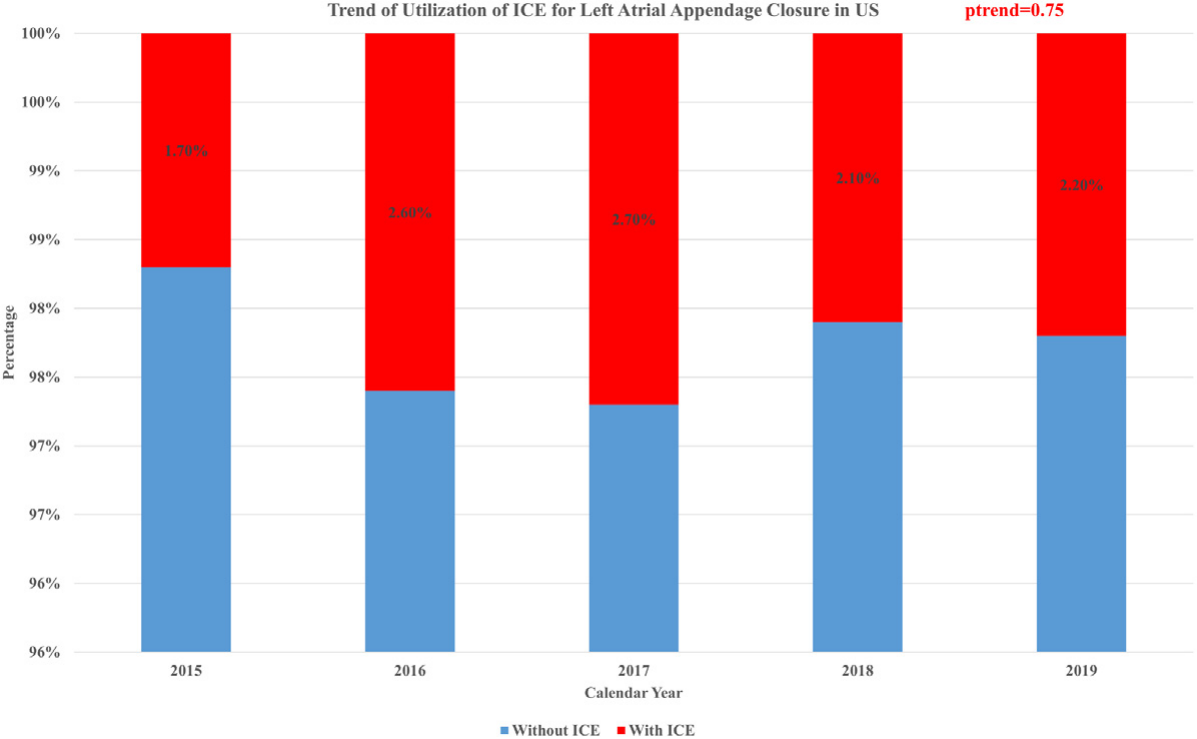
**Trend of ICE use for left atrial appendage closure in the US.** ICE, intracardiac echocardiography; US, United States.

During the study period from quarter 4 of 2015 to 2019, the use of ICE for LAAC remained low. The use of ICE guidance for LAAC increased from 1.7% in 2015 to 2.2% in 2019. The *P* value for temporal trend was not significant (*P*_trend_ = .75) (Figure 3).

There were no in-hospital deaths recorded in patients who had ICE-guided LAAC; however, the mortality difference between ICE-guided and TEE-guided cases was not significant (0% vs 0.2%; *P* = .14). Major complications, cardiovascular complications, neurologic complications, and pulmonary complications were similar for procedures performed under the guidance of ICE and TEE on both adjusted and unadjusted analyses. On crude analysis, ICE procedures had a higher rate of peripheral vascular complications (2.8% vs 1.3%), mainly driven by pseudoaneurysms (0.7% vs 0.3%) and retroperitoneal bleeding (0.7% vs 0.1%). On propensity-matched analysis, the overall vascular complication rates were similar in both groups; however, retroperitoneal bleeding remained significantly higher (<0.8% vs 0%) in the ICE-guided LAAC group. GI bleeding complications were more frequent in the TEE-guided LAAC group (3.5% vs 2.1%). Renal complications mainly driven by acute kidney injury were higher in the ICE group; however, after adjustment of comorbidities, the difference became nonsignificant (Table 2).

**Figure 4:**
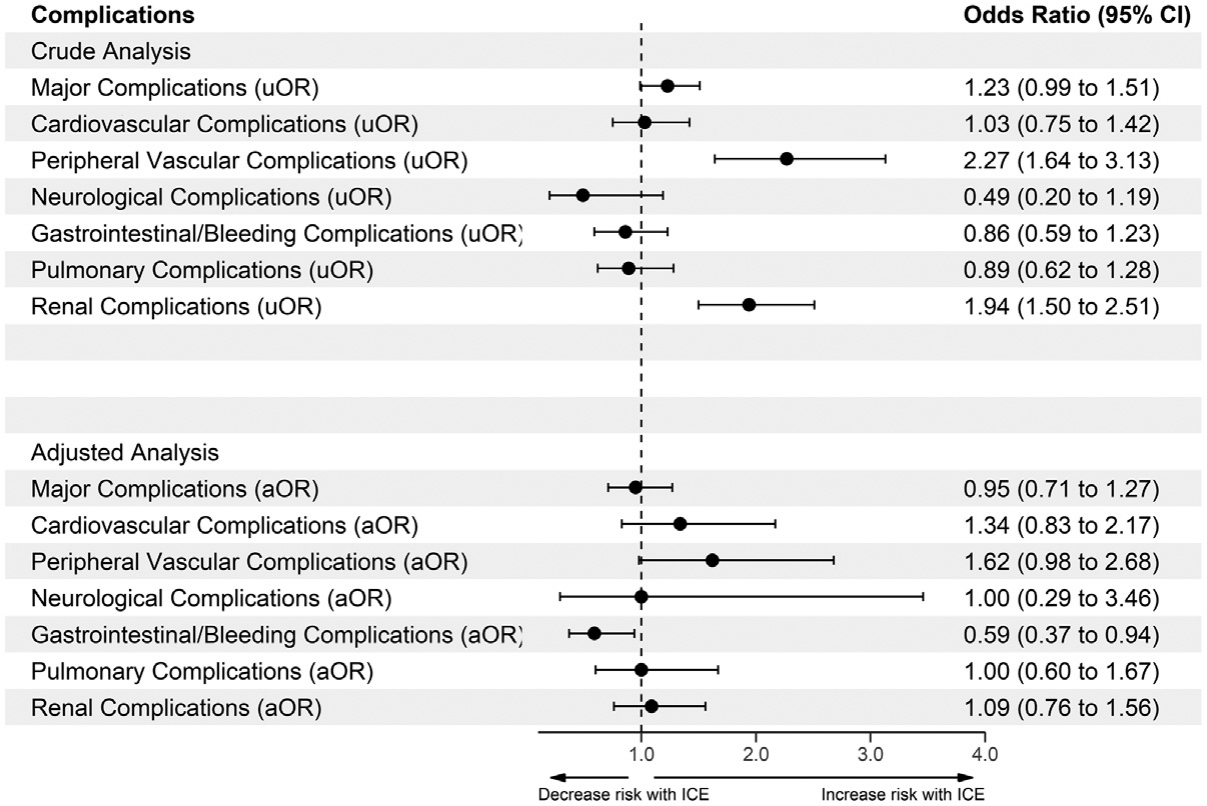
**Adjusted and unadjusted ORs for in-hospital complications.** aOR, adjusted odds ratio; ICE, intracardiac echocardiography; uOR, unadjusted odds ratio.

The unadjusted and adjusted propensity-matched ORs mirrored each other with the following exceptions. On unadjusted analysis, vascular complications were 2-fold higher in the ICE group (OR, 2.27; 95% CI, 1.64-3.13). However, in the propensity-matched sample, the difference between the 2 groups in terms of vascular complications was nonsignificant (OR, 1.00; 95% CI, 0.29-3.46). In addition, the odds of GI bleeding were significantly lower for ICE-guided LAAC than TEE-guided LAAC (OR, 0.59; 95% CI, 0.37-0.94) (Figure 4).

**Figure 5:**
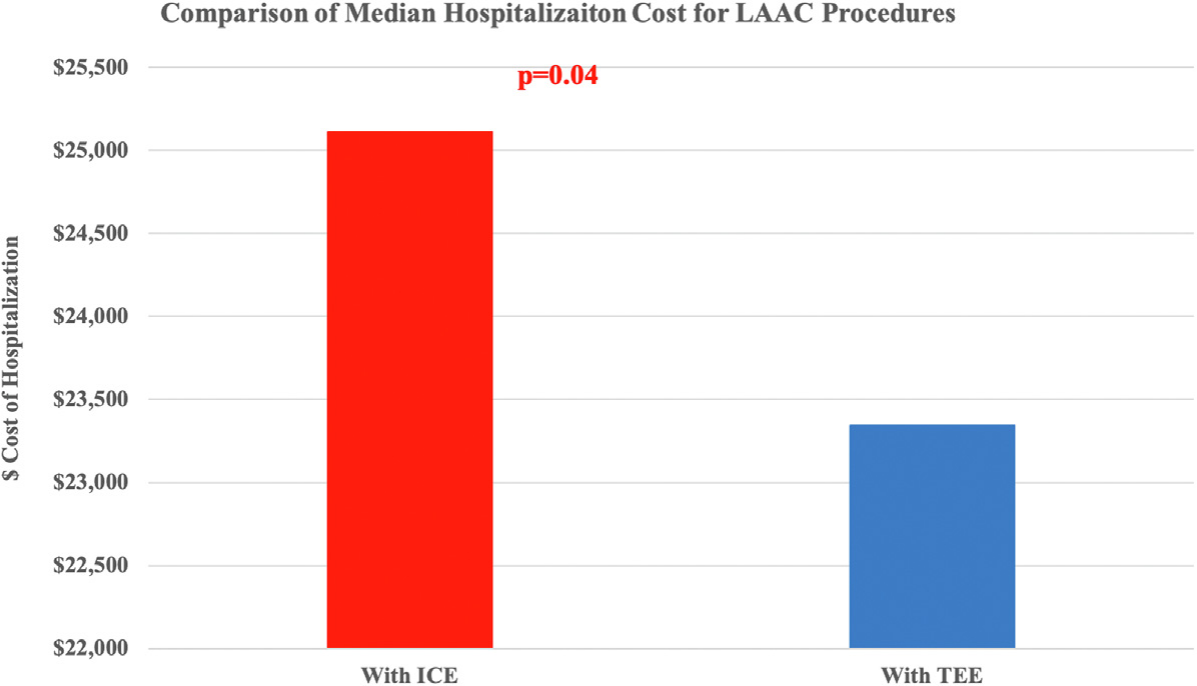
**Cost comparison for ICE-guided LAAC.** ICE, intracardiac echocardiography; LAAC, left atrial appendage closure; TEE, transesophageal echocardiography.

In terms of resource utilization, the length of hospitalization was similar for the 2 groups, with a median stay of 1 day (*P* = .23). In the propensity-matched sample, ICE use was associated with $1769 excess cost of hospitalization compared with TEE-guided LAAC ($25,112 vs $23,343; *P* = .04) (Table 2 and Figure 5).

## Discussion

This study analyzed the largest available database of hospitalizations in the United States from quarter 4 of 2015 to 2019, which included 61,995 LAAC procedures and 1410 ICE-guided LAAC cases. The key findings from our contemporary analysis are as follows:

1. Major complications, cardiovascular complications, neurologic complications, and pulmonary complications were similar for procedures performed with and without ICE guidance.
2. ICE-guided LAAC procedures are associated with lower GI bleeding complications but higher vascular complications.
3. The use of ICE for LAAC procedures remains low.
4. The length of hospitalization is comparable; however, the cost of hospitalization is higher when LAAC is performed under the guidance of ICE.

Prior studies evaluating the safety and efficacy of ICE for LAAC were mainly performed outside the United States.^6^^,^^11^^,^^12^ Hemam et al^13^ provided initial data from a multicenter and multioperator study (n = 104) supporting the safety of ICE-guided LAAC in the United States with the Watchman device. Alkhouli et al^8^ also supported the short-term safety of ICE-guided LAAC in a single-center study of 286 patients. However, large-scale studies evaluating the US national experience with ICE guidance for LAAC are lacking. Furthermore, the cost-effectiveness of this modality remains to be explored from a large database.

Our study shows a lower risk of GI bleeding complications in patients undergoing LAAC under the guidance of ICE. This is likely due to the avoidance of esophageal manipulation required with TEE.^14^ Acute kidney injury rates were higher among the patients in the ICE group in the unadjusted group, which could be due to the increased procedure time associated with ICE-guided procedures. Furthermore, respiratory failure and bronchospasm are also more common with TEE-guided procedures owing to the requirement of general anesthesia and endotracheal intubation.^15^ Our study complements these findings by reporting lower rates of respiratory complications in the ICE group. Conversely, vascular complication rates were higher with ICE. This is likely due to the requirement of additional venous puncture for vascular access.^16^ Overall, our study supports the safety of ICE use in LAAC by showing similar rates of major and cardiovascular complications and is concordant with prior studies.^8^^,^^13^

We found that the overall use of ICE for LAAC remained low in the United States and plateaued plated during our study duration. A similar trend for other structural heart disease interventions has also been observed, and it is postulated to be due to increased concerns about vascular complication rates, the cost of ICE catheters, lack of training, and a steep learning curve.^8^^,^^17^ Similarly, TEE may be preferable owing to the availability of Certified Registered Nurse Anesthetists in the United States and the adoption of micro TEEs.^18^ Although the use of ICE in the United States might be lower than in Europe, its use did increase significantly in the United States after 2019, which was not captured by our study. Our study reports higher adjusted costs ($1769 excess cost of hospitalization) in patients undergoing LAAC under the guidance of ICE. Previous studies have reported conflicting results on whether ICE use is associated with increased health care costs. The study by Isath et al^19^ reported that ICE use may be associated with a decreased length of hospital stay but an increased cost of hospitalization in patients undergoing atrial fibrillation ablation. In contrast, the study by Alqahtani et al^17^ reporting ICE outcomes with transcatheter aortic closure of interatrial communications showed similar hospitalization costs associated with ICE and TEE-guided procedures. Our study is concordant with the study by Alkhouli et al^8^ that showed that ICE use was associated with increased hospital charges. However, ICE guidance can save costs by avoidance of general anesthesia, potentially reprocessing ICE catheters, and eliminating the need for multiple personnel such as echocardiographers and anesthesiologists.^5^ Furthermore, with increased operator experience, LOS may decrease with ICE guidance and ultimately add to cost saving, although this needs to be systematically evaluated.

Our study adjusts for numerous known potential confounders; however, the impact of unknown and unmeasurable covariates could not be determined. Using the NIS selectively without any patient-level prospective data, the identified predictors may not include some covariates that may have eventually influenced LOS, cost of stay, and mortality. Because of the inherent limitations of cross-sectional data, no definitive conclusions regarding the causality of outcomes could be made. We were unable to conduct an individualized risk assessment. Because of a lack of well-validated *International Classification of Diseases* codes, we were not able to differentiate between upper or lower GI bleeds. Similarly, not all *International Classification of Diseases* codes used, such as that of retroperitoneal bleeding, have been validated by prior studies. Moreover, as NIS is a billing database, the possibility of errors due to undercoding or overcoding could not be entirely excluded. Furthermore, more recently, 3-dimensional and 4-dimensional ICE catheter technology is being used for structural intervention, which is expected to improve procedural outcomes by way of better imaging and intraprocedural guidance. Our study was not able to assess the utility of 3-dimensional ICE use because it was not available during the conduct of our study, and the coding does not identify the type of ICE catheter used in this database. ICE use increased during the COVID-19 pandemic, which was not captured by our study and is not reflective of the current state of practice. Furthermore, granular data on the efficacy of the procedure with respect to residual leaks at follow-up, procedure time, operator skill, and completion of closures are not available.

## Conclusions

In conclusion, ICE-guided LAAC is safer than TEE-guided LAAC, with similar rates of major complications. ICE use is associated with lower rates of GI bleeding; however, vascular complications such as retroperitoneal bleeding are higher. In addition, ICE-guided LAAC is associated with a similar LOS; however, the cost of hospitalization is higher compared with TEE-guided LAAC.
