# Supplementary material for: Feasibility and Safety of Intracardiac Echocardiography Use in Transcatheter Left Atrial Appendage Closure Procedures
Source: J Soc Cardiovasc Angiogr Interv. 2022 Nov 3;1(6):100510. doi: 10.1016/j.jscai.2022.100510 (PMC11307399; doi:10.1016/j.jscai.2022.100510)

SUPPLEMENTARY

# Supplementary Table S1: ICD-10 diagnosis and procedure codes used for the Study

| Variables | ICD-10 |
| --- | --- |
| Left atrial appendage closure | 02L73DK |
| Intracardiac Echocardiography | B244ZZZ, B245YZZ, B245ZZZ, B246YZZ and B246ZZZ |
| Transesophageal Echocardiography | B24BZZ4x |
| Coronary Artery Disease | I251, I257, I258, I259, I255 |
| Heart failure | I09.9, I11.0, I13.0, I13.2, I25.5, I42.0,  I42.5–I42.9, I43.x, I50.x, P29.0 |
| Peripheral vascular Disease | I70.x, I71.x, I73.1, I73.8, I73.9, I77.1,  I79.0, I79.2, K55.1, K55.8, K55.9,  Z95.8, Z95.9 |
| Cerebrovascular disease | G45.x, G46.x, H34.0, I60.x–I69.x |
| Chronic pulmonary  disease | I27.8, I27.9, J40.x–J47.x, J60.x–J67.x,  J68.4, J70.1, J70.3 |
| Diabetes Mellitus | E10.0, E10.1, E10.6, E10.8, E10.9,  E11.0, E11.1, E11.6, E11.8, E11.9,  E12.0, E12.1, E12.6, E12.8, E12.9,  E13.0, E13.1, E13.6, E13.8, E13.9,  E14.0, E14.1, E14.6, E14.8, E14.9 |
| Chronic Kidney Disease | N18 |
| End-Stage Renal Disease | Z992, N186 |
| Pulmonary circulation  disorders | I26.x, I27.x, I28.0, I28.8,  I28.9 |
| Peripheral vascular disorders | I70.x, I71.x, I73.1, I73.8,  I73.9, I77.1, I79.0,  I79.2, K55.1, K55.8,  K55.9, Z95.8, Z95.9 |
| Hypertension | I10.x |
| Liver disease | B18.x, I85.x, I86.4, I98.2,  K70.x, K71.1, K71.3–  K71.5, K71.7, K72.x–  K74.x, K76.0, K76.2–  K76.9, Z94.4 |
| Coagulopathy | D65–D68.x, D69.1, D69.3–  D69.6 |
| Obesity | E66.x |
| Weight loss | E40.x–E46.x, R63.4, R64 |
| Percutaneous coronary intervention | 02703, 02713, 02733 |
| Cardiac arrest | I46x |
| ST elevation myocardial infarction | I2101, I2102, I2109, I2111, I212, I2121, I2129 |
| Non-ST elevation myocardial infarction | I214 |
| Pericardial effusion requiring intervention | 0W9D3ZX, 0W9D3ZZ, 0W9D4ZX, 0W9D4ZZ, 0W9D40Z |
| Pericarditis | I300, I301, I308, I309 |
| Cardiogenic shock | R570, T8111XA |
| Anaphylaxis | T78 |
| Arterial thrombosis | I74 |
| Septic shock | R6521 |
| AV fistula | I770 |
| Pseudoaneurysm | I72 |
| Hematoma | L763 |
| Retroperitoneal bleeding | K661 |
| Venous thromboembolism | I82 |
| Hemorrhagic stroke | I60, I61, I62 |
| Ischemic stroke | I63 |
| Transient ischemic attack | G45 |
| Gastrointestinal bleeding | K92 |
| Bleeding during the procedure | L760, L761 |
| Need for blood transfusion | 3023. 3024, 3027 |
| Respiratory failure | J95x |
| Pneumothorax | J93 |
| Pleural Effusion | J90, J918 |
| Pneumonia bacterial | J15 |
| Need for a ventilator | 5A0935, 5A0945, 5A0955 |
| Acute kidney injury | N17 |
| New hemodialysis | Z4901 |

# Supplementary Table S2: Definition of primary and secondary outcomes

| Outcomes | Definitions |
| --- | --- |
| Major complications | Composite of pericardial effusion requiring intervention, cardiac arrest, ischemic stroke/ transient ischemic attack, hemorrhagic stroke, any bleeding, myocardial infarction and peripheral vascular complications which included AV fistula, pseudoaneurysm, access site hematoma, retroperitoneal bleeding and venous thromboembolism |
| Any cardiovascular complication | Composite of percutaneous coronary intervention, cardiac arrest, heart block, ST elevation myocardial infarction, non-STE elevation myocardial infarction, pericardial effusion requiring intervention, pericarditis, and cardiogenic shock |
| Any systemic complications | Composite of anaphylaxis, arterial thrombosis, septic shock |
| Any peripheral vascular complication | Composite of arteriovenous fistula, pseudoaneurysm, hematoma, retroperitoneal bleeding, and venous thromboembolism |
| Any neurological complication | Composite of Hemorrhagic stroke, Ischemic stroke and transient ischemic attack |
| Any gastrointestinal or hematological complication | Composite of gastrointestinal bleeding, bleeding during the procedure and need for blood transfusion |
| Any pulmonary complication | Composite of respiratory failure, pneumothorax, pleural effusion, bacterial pneumonia and need for mechanical ventilation |
| Renal complications | Acute kidney injury and new hemodialysis |

#

**Supplementary Figure S1**


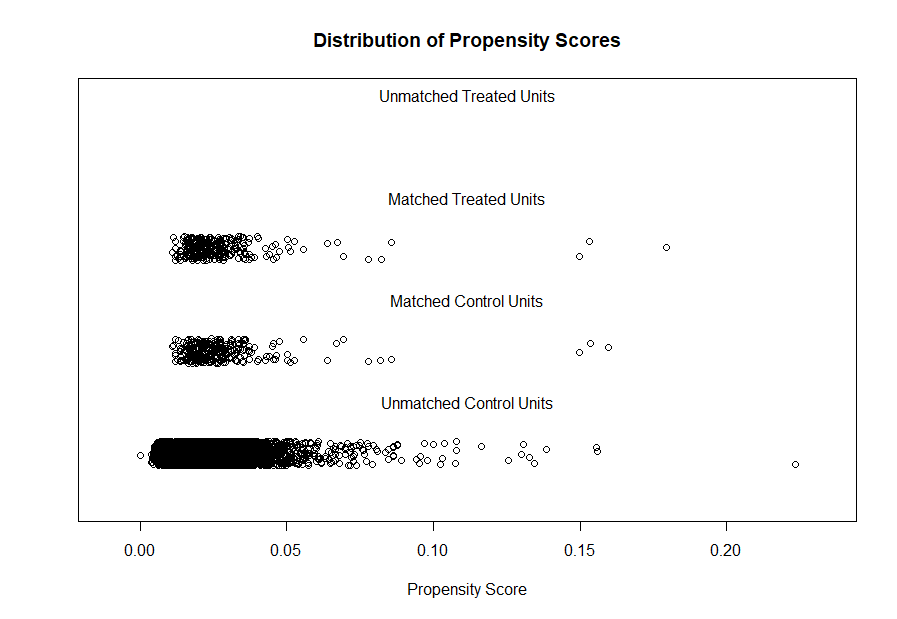


**Supplementary Figure S2**


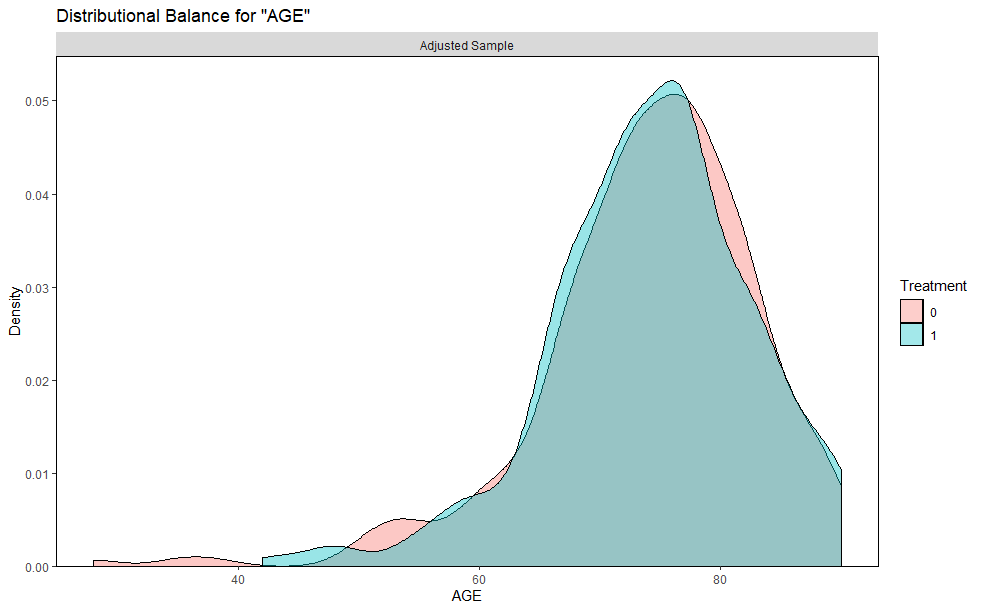


**Supplementary Figure S3**


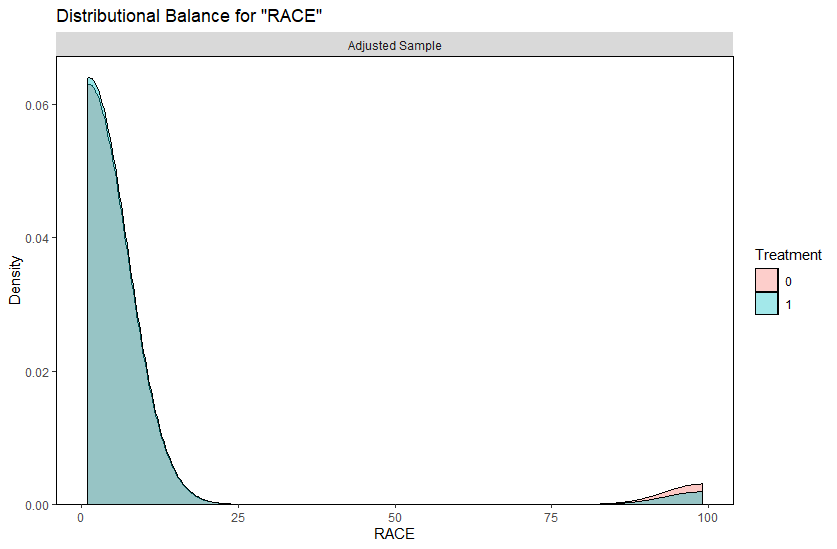


**Supplementary Figure S4**


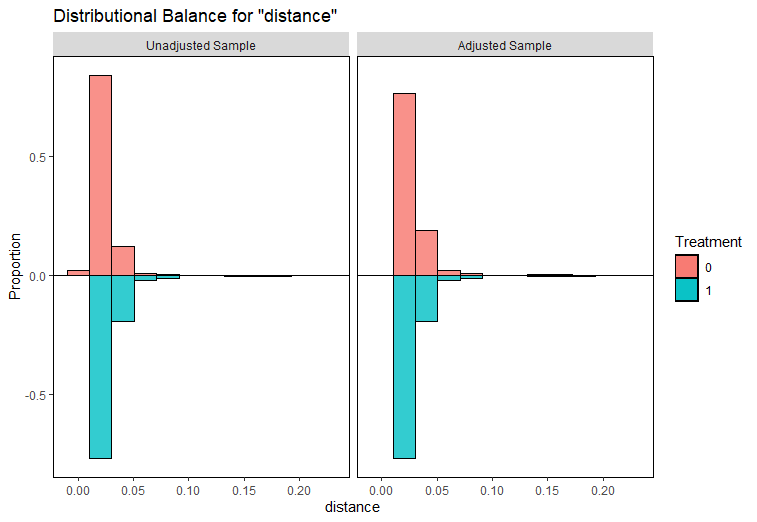

Supplement: Supplementary [file mmc1.docx]
